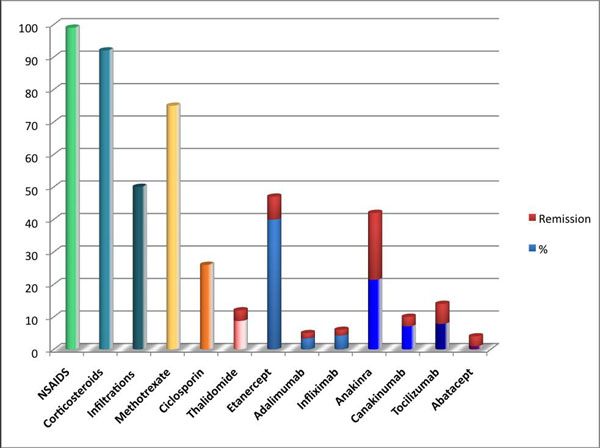# Systemic onset Juvenile Idiopathic Arthritis (SoJIA): a monocentric study of 114 patients

**DOI:** 10.1186/1546-0096-9-S1-P178

**Published:** 2011-09-14

**Authors:** I Melki, J Djadi-Prat, AM Prieur, C Job-Deslandre, P Quartier, C Elie, B Bader Meunier

**Affiliations:** 1 Unité d’Immunologie, hématologie et Rhumatologie pédiatrique, Hôpital Necker Enfants malades, 75015 Paris, France; 2 Service de biostatistiques, Hôpital Necker Enfants malades, 75015 Paris, France

## Background

SoJIA is a rare paediatric disease, which may evolve in monophasic, polycyclic and chronic persistent course. Only a few studies have tried to bring out early factors of poor outcome that could lead to manage SoJIA according its severity at onset.

## Aim

To describe presentation and outcome of SoJIA and to determine early clinical and laboratory characteristics associated to a poor outcome.

## Methods

Retrospective study on SoJIA cases diagnosed by ILAR criteria between January 1985 and December 2005 in a tertiary paediatric rheumatology centre.

## Results

During the study period, 114 children were included. Mean age at diagnosis was 4 years and 4 months (range, 4 months to 15.5 years). Familial history of autoimmunity was found in 27% of the patients. Glycosylated ferritin was low (≤20%) for 79% of patients. Twelve percent of patients had macrophage activation syndrome, 2 patients anti-neutrophil cytoplasmic antibodies (ANCA) associated glomerulonephritis, 3 patients pulmonary restrictive syndrome without vertebral arthritis, and one patient Crohn’s disease; 11 % had positive antinuclear antibody (ANA) (≥1/160) at diagnosis. None developed neoplasia. Patients in whom diagnosis was made after 2000, and had been treated by biotherapy, especially anakinra, had less osteoarticular sequelae and lower inflammatory syndrome (p = 0,004) than others (Figure 1). Early polyarticular and wrists involvements were associated to a poor outcome (p = 0,009, p = 0,027).

## Conclusion

This retrospective study suggests that 1) autoimmunity might be involved in the pathogenesis of SoJIA 2) early polyarticular and wrist involvements are associated with a poor outcome 3) anti-IL1 agents’use is associated with an improvement of the outcome.

**Figure 1:**